# Salmonella bloodstream infection and concurrent intestinal colonization in children with severe acute malnutrition in Niger

**DOI:** 10.1099/mgen.0.001788

**Published:** 2026-07-20

**Authors:** Kirsty Sands, Raúl Campillo, Ian Boostrom, Giulia Lai, Aditya Kumar Lankapalli, Kate Cook, Brekhna Hassan, Edward AR Portal, Mei Li, Lim S. Jones, Tassiou Elhadji Ibrahim, Idi Kalla, Nathan Sayinzonga-Makombe, Sheila Isanaka, Rupa Kanapathipillai, Christopher Mambula, Isabelle Mouniaman, Céline Langendorf, Timothy R Walsh, Owen Brad Spiller

**Affiliations:** 1 Division of Infection and Immunity, Cardiff University, Cardiff, UK; 2 Ineos Oxford Institute for Antimicrobial Research, Department of Biology, University of Oxford, Oxford, UK; 3 Departamento de Producción Animal y Ciencia de los Alimentos, Facultad de Veterinaria, Instituto Agroalimentario de Aragón-IA2 (Universidad de Zaragoza-CITA), Zaragoza, Spain; 4 Public Health Wales Microbiology, University Hospital of Wales, Cardiff, UK; 5 Ministry of Public Health and Hygiene, Niamey, Niger; 6 Epicentre, Maradi, Niger; 7 Department of Research, Epicentre, Paris, France; 8 Department of Nutrition, Harvard T.H. Chan School of Public Health, Boston, Massachusetts, USA; 9 Médecins Sans Frontières, Paris, France

**Keywords:** *Salmonella*, bloodstream infection, rectal carriage, Niger, genomic relatedness

## Abstract

Children with severe acute malnutrition (SAM) are at high risk of invasive infection. In sub-Saharan Africa, invasive non-typhoidal *Salmonella enterica* (iNTS) serovars are a leading cause of paediatric bloodstream infection. However, low-resource settings lack genomic data linking intestinal carriage with concurrent BSI in children presenting with SAM.

We conducted a longitudinal cohort study among children aged 0–59 months admitted for inpatient management of complicated SAM at the Madarounfa Intensive Nutritional Rehabilitation Centre, Maradi, Niger (2016–2017). Blood cultures and rectal swabs were obtained on admission (and during hospitalization if symptoms worsened). Genomic population structure of *S. enterica* serovars Enteritidis and Typhimurium was compared using phylogeny and clustering approaches. Among 1,371 enrolled children, 87 *Salmonella* isolates were recovered from 83 BSI episodes in 80 children. Invasive isolates belonged to a few globally circulating lineages, whereas carriage-only isolates demonstrated more heterogeneous genotypes. Minimum inhibitory concentrations were determined for 58 viable BSI isolates: susceptibility was retained to third-generation cephalosporins and carbapenems, while resistance to ampicillin (95%) and amoxicillin/clavulanate (90%) was common.

A nested case–control analysis of rectal swabs from 232 children (58 iNTS BSI cases; 174 non-BSI controls) was performed to test the association between gut carriage and BSI. *Salmonella* was detected in 44/58 (76%) cases vs. 7/174 (4%) controls, yielding a relative risk of 18.86 (CI 95% 9.00–39.53; *χ*²=126.7; *P*<0.0001). Clustering analyses confirmed close genomic relatedness within patient pairs and indicated circulation of a limited number of endemic clones. Among controls with faecal *Salmonella*, serovars were diverse and distinct from invasive lineages.

**Impact Statement**

We enrolled 1,371 children admitted for inpatient severe acute malnutrition (SAM) care in Niger and collected blood cultures and rectal swabs. When children with SAM present to hospital, it is unclear whether *Salmonella* in the gut is linked to the bacteria invading the bloodstream. Children with *Salmonella* bloodstream infection were ~19 times more likely to carry *Salmonella* in their rectum than children without BSI (relative risk 18.86; *P*<0.0001). Intestinal carriage was strongly associated with and often genomically comparable to the invasive bloodstream strain at presentation. In complicated SAM, the gut could be a key reservoir for invasive *Salmonella* at the time of illness, with a small number of successful clones driving most disease. Surveillance and interventional strategies including microbiome-focused measures may reduce reinfection and transmission.

## Data Availability

All genomes have been uploaded to National Center for Biotechnology Information (NCBI) and assigned the project accession number PRJNA1096457 and S3 Data lists all individual accession numbers.

## Introduction

Undernutrition has serious health consequences, contributing to up to 45% of deaths in children under 5 [1,2], with the highest burden in Africa and South Asia. Severe acute malnutrition (SAM) is defined as a weight-for-height *z*-score of less than −3 or a mid-upper arm circumference <115 mm [3,4]. Uncomplicated SAM can be managed in the community with ready-to-use therapeutic food [5]. The World Health Organization (WHO) recommends an oral course of amoxicillin for children with uncomplicated SAM, as many present with subclinical symptoms [6,7]. By contrast, SAM with complications such as infection or metabolic dysfunction usually requires hospitalization, as well as intravenous or oral antibiotics (e.g. amoxicillin, gentamicin and metronidazole) [8,9]. Infection risk is high in SAM due to impaired immunity, with ~15% of affected children requiring hospitalization to stabilize clinical complications. Broad-spectrum *β*-lactam antibiotic usage is high due to a large burden of infectious disease and is part of the recommended treatment for complicated SAM. Repeated exposure to antibiotics in the first years of life may consequently disrupt gut microbiome [10,12] and promote intestinal carriage of multidrug-resistant (MDR) *Enterobacterales* [13,14].

*Salmonella* infections are a major global concern. *Salmonella enterica* (>2,600 serovars) includes both typhoidal and non-typhoidal serovars (NTS) [15,16]. NTS frequently cause bloodstream infections (BSI) in immunocompromised hosts and are a leading cause of invasive bacterial disease in sub-Saharan Africa, where cases are termed invasive NTS (iNTS) [17,18]. Salmonellosis typically presents with diarrhoea and bacteraemia but can also involve other organs, especially in the context of co-infections [16]. Pathogenicity varies by serovar and host status, yet *Salmonella* consistently ranks among the top three causative BSI pathogens in low- and middle-income countries [19,22].

Globally, SAM and related nutritional disorders disproportionately affect children, especially in Africa and South Asia [4,9]. Drivers include poverty; poor access to water, sanitation and hygiene (WASH); and climate variation, with many sub-Saharan countries experiencing seasonal peaks in SAM [23,28]. In this study, we aimed to [1] characterize the genomic diversity of *Salmonella* causing BSI in children admitted to an inpatient SAM treatment facility building on previous clinical microbiological and epidemiological data published [2,29] test whether children with *Salmonella* BSI were more likely to carry *Salmonella* in the gut and [3] examine genomic relatedness between invasive and carriage isolates to explore potential links between colonization and systemic infection.

## Methods

### Study design

A longitudinal study was conducted between September 2016 and December 2017 at the Madarounfa Intensive Nutritional Rehabilitation Centre (CRENI), Maradi, Niger, managed by the Ministry of Public Health with support from Médecins Sans Frontières. Children aged 1–59 months admitted without the need for emergency care were enrolled in the study. Informed consent was obtained from caregivers, and the full study design has been described elsewhere [29]. To assess the association between *Salmonella* carriage and BSI, we performed a retrospective nested case–control analysis within the cohort. Controls were selected at a 3:1 ratio to cases across the enrolment period [30], with frequency-unmatched selection by age and sex distribution.

### Sample collection

Blood samples were collected and cultured in Niger on admission for children presenting with sepsis or infection. Blood cultures were also collected during the course of treatment in the case of clinical deterioration. For all children enrolled in the study, rectal swabs were taken on admission, during hospitalization and at the same time as blood cultures if clinical deterioration occurred and at discharge. Blood samples were inoculated into BacT/ALERT PF paediatric FAN bottles and incubated aerobically for up to 7 days [29]. Gram staining and API strips (bioMérieux) were used for identification of positive cultures. Blood culture isolates were preserved on charcoal swabs; rectal swabs were stored at 4–8 °C and shipped to Cardiff University.

### BSI culture and antibiotic susceptibility testing

Isolates preliminarily identified as *Salmonella* Typhi were sent to the UK Health Security Agency (UKHSA) for confirmation and whole-genome sequencing (WGS). At Cardiff University, NTS were cultured on chromogenic agar with vancomycin (10 mg l^−1^) and xylose lysine deoxycholate (XLD) (Sigma) agar, incubated overnight at 37 °C, and identified by MALDI-TOF MS (Bruker). Minimum inhibitory concentrations (MICs) for 19 antibiotics (ampicillin, cefepime, amikacin, ceftazidime, cefotaxime, ertapenem, amoxicillin/clavulanate, ceftazidime/avibactam, ciprofloxacin, fosfomycin, meropenem, ceftriaxone, colistin, imipenem, levofloxacin, gentamicin, tobramycin, piperacillin/tazobactam and aztreonam) were determined by agar dilution [31]. *Escherichia coli* ATCC 25922 and a PBS blank were used as controls, and results were interpreted using EUCAST v15.

### Rectal swab processing

Rectal swabs were collected on patient admission, during hospital stay [in the case of deterioration and/if a healthcare-associated infection (HAI) was suspected] and on discharge. Swabs were screened for *Salmonella* using XLD agar (Sigma) and incubated overnight at 37 °C. Colonies with characteristic morphology were sub-cultured and identified by MALDI-TOF MS. Up to five colonies per sample (more if phenotypically diverse) were processed. All confirmed *Salmonella* were genotyped by repetitive element sequence-based PCR (REP-PCR), and representative REP types were selected for WGS for comparison to corresponding BSI isolates.

### REP-PCR molecular typing to assess BSI and carriage isolate relatedness

NTS isolates from blood cultures were compared to phenotypically matching rectal isolates. REP-PCR was performed on all BSI isolates and up to five rectal colonies per patient. The primers REPF-RI (5′-IIIGCGCCGICATCAGGC-3′) and REPR-2I (5′-ICGICTTATCIGGCCTAC-3′) were used in 25 µl reactions, under the following conditions: 95 °C 7 min; with 30 cycles of 90 °C 30 s, 40 °C 1 min, 65 °C 8 min; and a final extension of 65 °C 16 min. Amplicons were visualized by 2% agarose gel electrophoresis at 240 V for 150 min. Profiles were clustered by unweighted pair group method with arithmetic mean. Isolates with ≥88% similarity [32,33] to corresponding BSI isolates were sequenced, alongside all BSI isolates.

### Whole-genome sequencing

Between 2017 and 2019, all BSI isolates underwent short-read WGS using Nextera XT for library preparation and paired-end sequencing (v3, 2×300 bp) on an Illumina MiSeq as described elsewhere [34]. *S*. Typhi isolates were sequenced at UKHSA (HiSeq). Additionally, 22 rectal isolates were sequenced as described above based on matching REP-PCR profiles, and four of these rectal carriage isolates, each representing the major lineages observed, were selected for long-read sequencing using Nanopore Rapid Barcoding (SQK.RBK004) and were sequenced on R9.4 flow cells (run for 72 h) using a MinION device (Oxford Nanopore Technologies, UK). Basecalling was performed using Guppy (v5.0.11) within MinKnow. In 2024, 23 archived isolates were sequenced using a GridION device (FLO-MIN114 R10.4.1 flow cells, SQK-RBK110.96), with high-accuracy basecalling performed using Guppy v7.2.13. The 23 isolates were selected to provide higher-resolution genomic comparison in cases where a bloodstream infection (BSI) isolate and a rectal carriage *Salmonella* isolate from the same child shared the same ST, or where a rare serovar was identified in rectal carriage isolates.

### Bioinformatics analysis

Short reads were trimmed using TrimGalore v0.6.10 [35], assembled using Shovill v0.9.0 [36] and contigs <200 bp were removed using Seqtk v1.3 [37]. Assemblies were quality-checked with QUAST v5.2.0 [38], and poor-quality genomes defined as those with ±10% expected genome size or those with >1,000 contigs were excluded. Serovar was confirmed using sistr v1.1.3 [39]. Genomes were screened for antimicrobial resistance genes (ARGs) using AMRfinderPlus v3.12.8 (database version 2024-01-31.1) [40]. ABRicate v1.4.0 [41] and associated databases PlasmidFinder (April 2026) [42] and VFDB (Apr 2026) [43] were used to screen genomes for plasmid incompatibility types and virulence-associated genes. Multilocus sequence type (MLST) was identified using mlst v2.23.0 [44], and *S*. Typhi clades were assigned using Genotyphi [45] in PathogenWatch v23.4.1. Genomes were annotated with Bakta v1.9.4 db v5.1 [46], and a core genome alignment was performed with Panaroo v1.5.2 [47]. A maximum-likelihood core genome phylogenetic tree was inferred using IQ-TREE v2 with 1,000 bootstrap replicates [48] and visualized in iTOL v7 [49].

Rectal carriage isolates with short- and long-read sequencing data (*n*=4) were hybrid-assembled using Unicycler v0.5.0 [50]. Nanopore-only long reads (2024–2025) were trimmed using nanoq v0.10.0, assembled with Flye v2.9.0 [51] and polished with Medaka v1.12.1. Genome annotation, MLST, ARG and virulence-associated genes were performed as described for BSI isolates. For genomes containing long reads, mobsuite v3.1.9 [52] was used to reconstruct and assemble plasmids. Reconstructed plasmids were assessed for the presence of virulence-associated, antibiotic resistance and plasmid incompatibility genes using ABRicate v1.4.0 and associated databases as described above. The presence of virulence-associated genes/determinants was compared between rectal carriage and BSI *Salmonella* isolates.

To determine genomic similarity between BSI and rectal carriage isolates, *S*. Typhimurium and *S*. Enteritidis genomes were clustered with PopPUNK v2.6.3 [53], with reference genome collections obtained from (NCBI) National Center for Biotechnology Information (downloaded July 2024). A total of 30,000 genomes were downloaded from NCBI, which were sub-sampled to 5,000 using MLST v2.23.0 to maximize diversity. Genomes with >1,000 contigs were excluded. For each serovar independently, genomes were sketched and clustered using PopPUNK to infer pairwise core and accessory genome distances. Following model comparison to determine appropriate models according to scale and diversity of each dataset, HDBSCAN and BGMM models were used to cluster *S*. Enteritidis (1,280 NCBI genomes used to fit the model) and *S*. Typhimurium (2,579 NCBI genomes used to fit the model), respectively (Figs S1 and S2). Genomic clusters were visualized with GrapeTree v1.5.0 [54]. SNP analysis was performed to determine genomic distances between rectal carriage and BSI isolates detected from within the same patient using snippy (v4.6.0) at default parameters. A local reference for each ST11 and ST7945 *S*. Enteritidis and ST313 *S*. Typhimurium was selected from the available hybrid genomes (Data S3) [55]. Snippy-core was performed for each sequence type (ST) group, and a pairwise SNP matrix was generated using snp-dists (v1.2.0) [56].

### Statistical analysis

Two assumptions guided analysis: (i) controls with negative blood cultures on admission were assumed negative for BSI, and (ii) rectal samples negative on XLD were considered *Salmonella*-free. Due to biosafety reasons, rectal samples from *S*. Typhi BSI cases could not be processed, so controls only matched iNTS cases. Relative risks and 95% confidence intervals were calculated from 2×2 contingency tables, and the association between intestinal iNTS carriage and BSI was tested using chi-squared tests with Yates correction. Analyses were performed in RStudio v2024.12.1+563 with *epitools*, *ggplot2*, *ggmosaic* and *cowplot*. Figures were generated using *fuzzysim*, *AMR*, *ggplot2*, *readxl*, *dplyr*, *forcats, patchwork* and *pheatmap*.

## Results

### Cohort characteristics and BSI onset

**Fig. 1.**
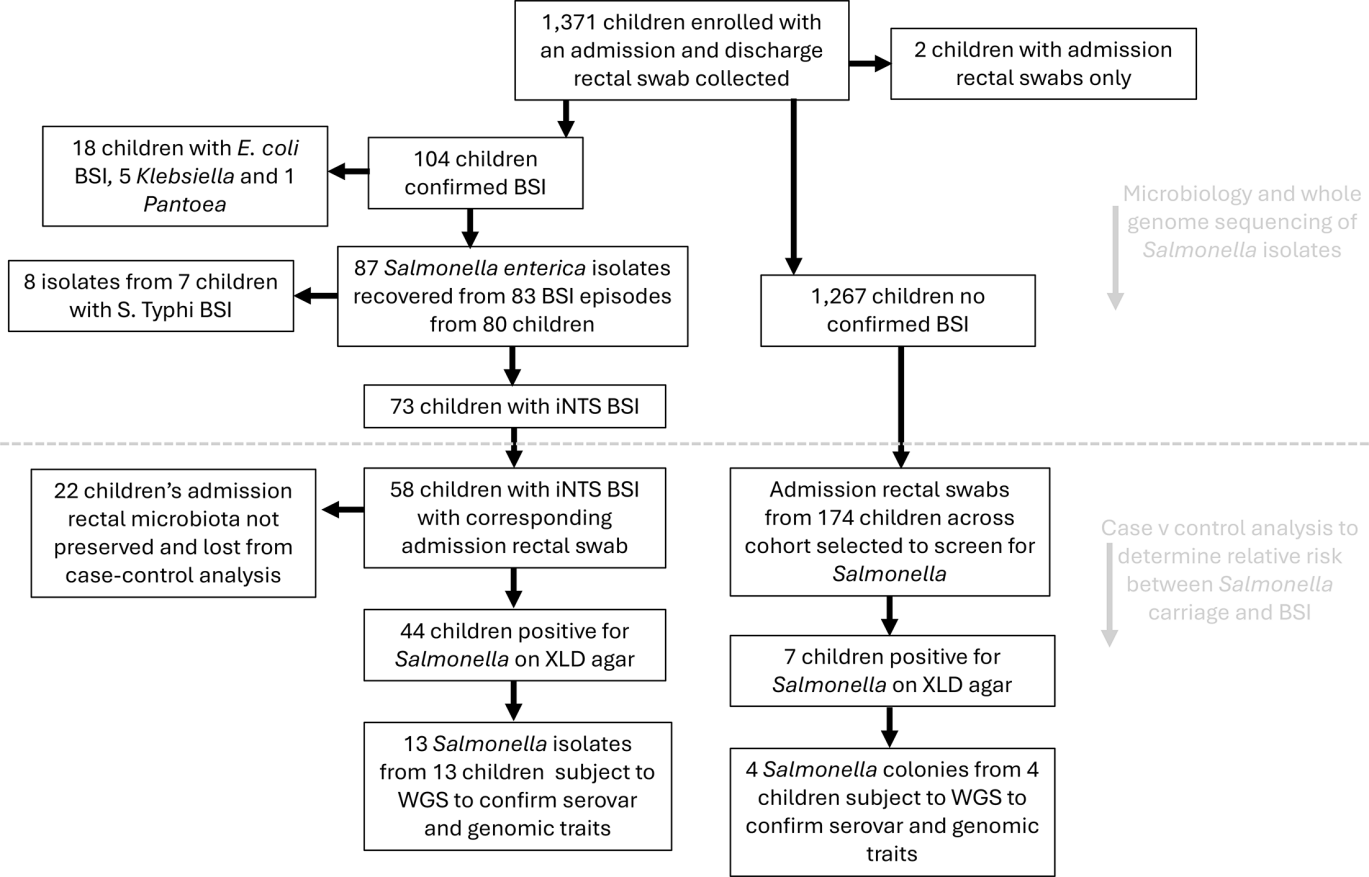
Study STROBE flow diagram illustrating participant enrolment, sample collection and analysis through each phase of the study.

Between September 2016 and December 2017, 1,371 children (aged 1–59 months) with complicated SAM were enrolled in the study (Fig. 1, Data S1 (available in the online Supplementary Material). Rectal swabs were collected at admission and discharge for 1,369 children (99%), with additional swabs during hospitalization if deterioration occurred [31] (Fig. 1, Data S1). *Salmonella* were isolated from 87 blood cultures from 80 children (83 BSI episodes, Fig. 1, Table 1) [29]. Four duplicate isolates were recovered from repeat cultures. Of these, 74/83 (89%) were community-acquired infection (CAI) positive on admission (67 iNTS, 7 *S*. Typhi), and 9 were HAI, a positive blood culture after 48 h of hospitalization. Three patients presented with community-acquired (CA) BSI on admission and later developed a second healthcare-associated (HA) BSI with either a different serovar or ST (Table 1). Niger has a short rainy season (June–September) and a long dry season (October–May). Although enrolment spanned 16 months, seasonal variation was difficult to assess; however, 35% (28/80) of children with *Salmonella* BSI were admitted in October (start of dry season aligning with annual malaria and malnutrition peaks), with peaks in both 2016 (13 children, 14 isolates) and 2017 (15 children, 25 isolates).

**Table 1.** Bloodstream infection cohort summary characteristics split by *Salmonella* serovar

	*S. enterica* subsp. *enterica* serovar Colindale	*S. enterica* subsp. *enterica* serovar Enteritidis	*S. enterica* subsp. *enterica* serovar ND	*S. enterica* subsp. *enterica* serovar Typhimurium	*Salmonella* Typhi
Number of isolates	1	47	3	28	8
Number of patients with *Salmonella* serovar BSI*	1	45	3	27	7
Patient sex	*n*=1 F	*n*=20 F, *n*=25 M	*n*=2 F, *n*=1 M	*n*=11 F, *n*=17 M	*n*=4 F, *n*=4 M
Acquisition (CA vs. HA)	*n*=1 A	*n*=39 CA, *n*=6 HA	*n*=3 HA	*n*=23 CA, *n*=5 HA	*n*=7 CA, *n*=1 HA
Temporal recovery across enrolment	–	13 months	All distinct	13 months	11 months
ST	*n*=1 ST4216	*n*=27 ST11, *n*=2 ST1479, *n*=18 ST7945	nd	*n*=27 ST313, *n*=1 ST7949	*n*=8 ST2

*n=3 patients had BSI with>1 *S. enterica* serovar identified.

### *S. enterica* BSI

**Fig. 2.**
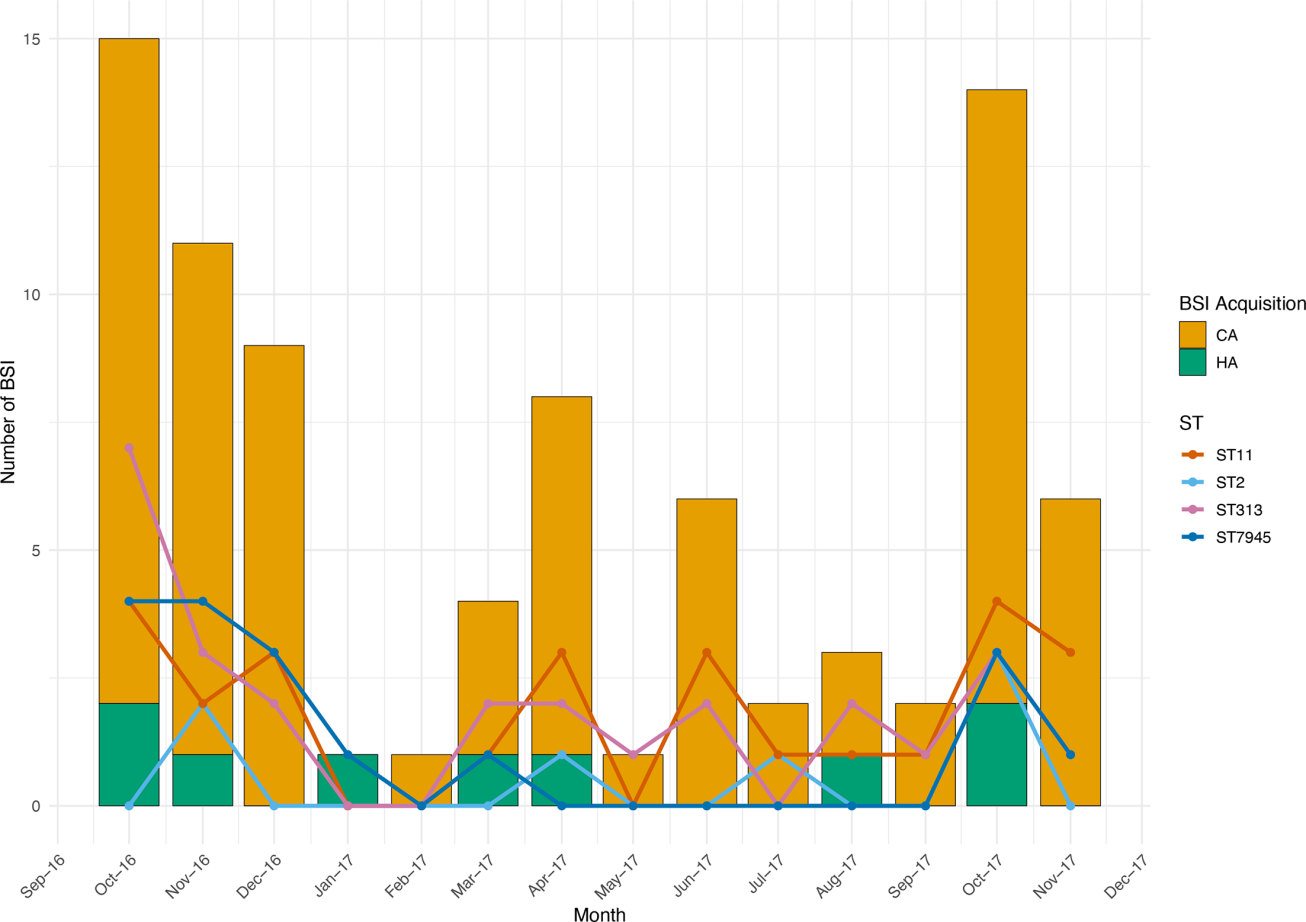
Monthly distribution of BSI by acquisition setting and dominant ST, October 2016–November 2017 (no BSI recorded in September 2016, enrolment began on 26 September 2016); over time, double plot. Stacked bars show the number of CA and HA BSIs per month. Overlaid lines represent the monthly counts of four predominant sequence types (ST2 *Salmonella* Typhi, ST11 *Salmonella enterica* subsp. Enteritidis, ST313 *Salmonella enterica* subsp. Typhimurium and ST7945 *Salmonella enterica* subsp. Enteritidis).

**Fig. 3.**
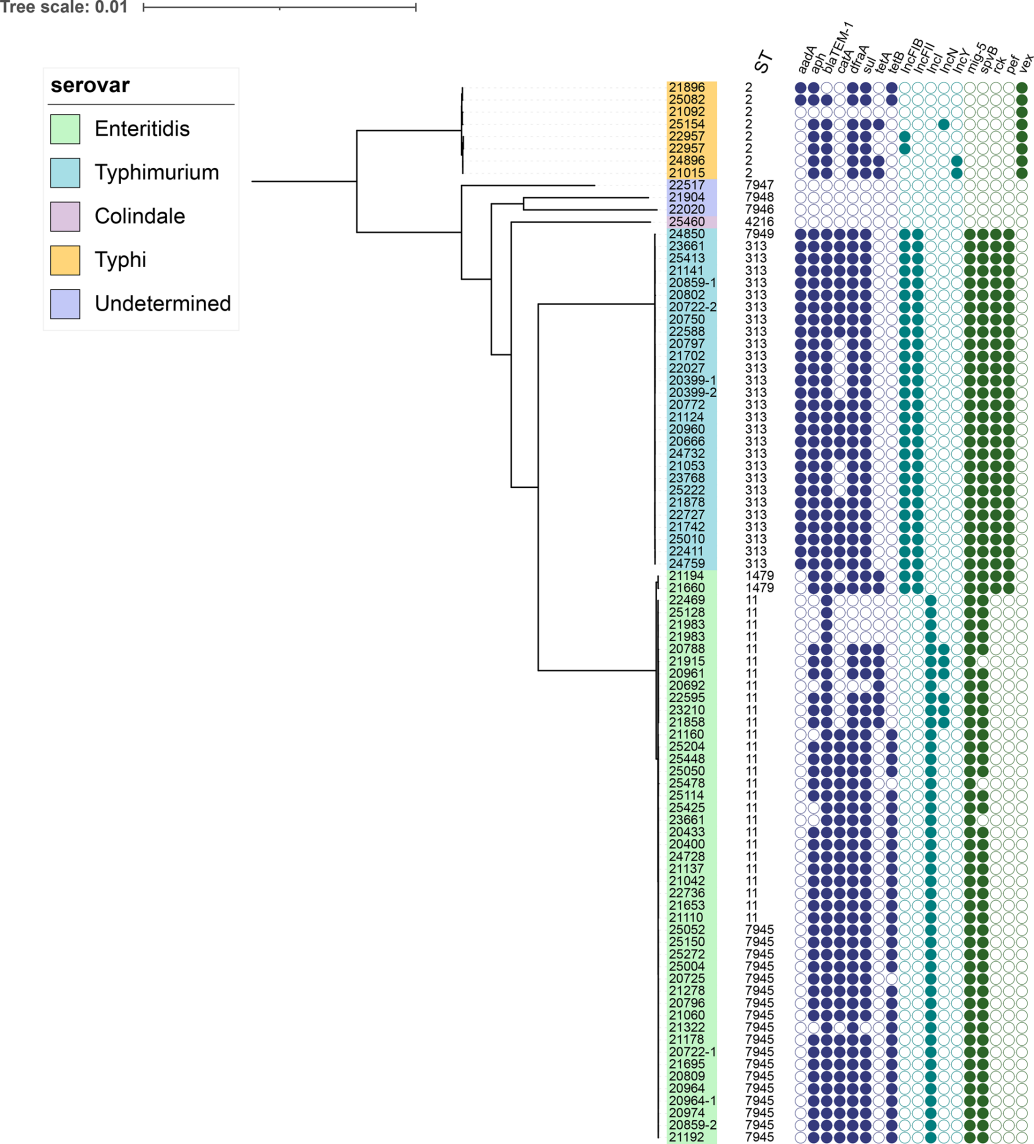
Core genome phylogenetic tree (98% core threshold used) for *S. enterica* (*n*=87) bloodstream infection genomes listing the three major serovars identified. The ST follows the patient ID and BSI isolate number. A heat map represents identified ARGs, virulence-associated genes and plasmid incompatibility types.

WGS confirmed 47 *S*. Enteritidis, 28 *S*. Typhimurium, 1 *S*. Colindale, 3 undetermined and 8 *S*. Typhi isolates (Figs2  3, Data S2). Of 87 total isolates, 79 were iNTS. Ten STs were identified; most prevalent were *S*. Typhimurium ST313 (*n*=27), *S*. Enteritidis ST11 (*n*=27) and *S*. Enteritidis ST7945 (*n*=18; assigned in this study). ST7945, a single-locus variant of ST11, may have evolved locally and co-circulated with ST11, re-emerging during the 2017 dry season (Table 1, Fig. 2). All *S*. Typhi BSI were CA; isolates were ST2 (Fig. 3), clonal complex 13, with genotypes spanning clades 2.2 (*n*=2), 2.3.1 (*n*=2) and 3.1.1 (*n*=4), indicating diversity despite identical STs.

### Antimicrobial resistance

**Fig. 4.**
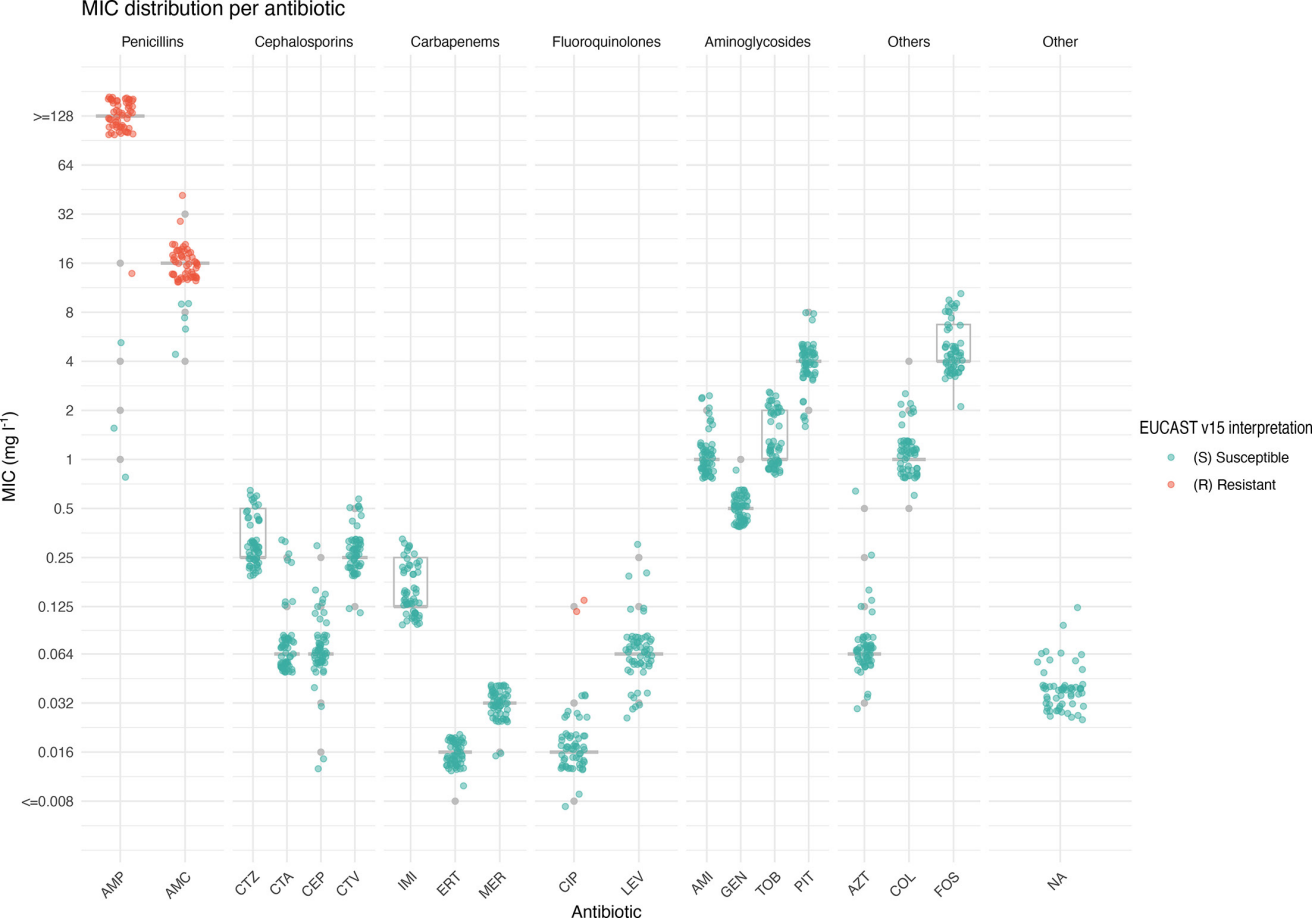
MIC distributions for 19 antibiotics tested against *S. enterica* bloodstream infection isolates. Where applicable, box plots display the range and median MIC values. Individual data points are coloured by their susceptibility interpretation according to EUCAST v15 guidelines: susceptible (S) and resistant (R). Antibiotics are grouped and ordered by their antimicrobial class for clarity.

MICs were determined for 58 re-cultured isolates (Fig. 4). No significant differences were observed between CAI and HAI BSI; however, comparisons were limited as there were only 11 HAI isolates with available MIC data (Data S2). All iNTS isolates were susceptible to aminoglycosides (however clinical guidance recommends the interpretation of aminoglycosides resistant irrespective of *in vitro* testing outcomes), cephalosporins and carbapenems (Fig. 4). Resistance was high to ampicillin (95%, 55/58) and co-amoxiclav (90%, 52/58), with *bla*_TEM-1_ found in all cases where phenotypic resistance to ampicillin and co-amoxiclav was observed and in 80/87 genomes overall (Fig. 3). Ciprofloxacin resistance was rare (3%, 2/58) caused by a somatic mutation in *gyrA-D87Y*; *qnrA*, *qnrB*, *qnrS* or *aac(6′)-Ib-cr* were not detected in the genomes. However, levofloxacin susceptibility was 100%, including the two isolates resistant to ciprofloxacin. Trimethoprim resistance genes (*dfrA1*, *dfrA7* and *dfrA14*) were detected in 76 genomes, often reported to be plasmid-associated [57] (Fig. 3). ARGs were carried on conjugative IncF, IncI and IncN plasmids (Fig. 3, Data S3). Although MICs were not determined for *S*. Typhi, associated resistance genes except chromosomal *aac(6)* were located on sequences identified as plasmid DNA when the contig was analysed. Predicted resistance was determined in 6/8 isolates to ampicillin (*bla*_TEM-1_), in 7/8 to sulphonamides/trimethoprim (*sul/dfrA* genes) and in 5/8 to tetracycline (*tetA/tetB*). Ciprofloxacin was susceptible at increased exposure for three isolates that all contained *gyrA-S83Y* or *gyrB-S464F* mutations. Short-read data limited *S*. Typhi plasmid analysis, but incompatibility typing identified IncY/IncFIB in genotype 3.1.1, IncN in genotype 2.2 and no plasmids in genotype 2.3.1 (Fig. 3, Data S3).

### Virulence-associated determinants

**Fig. 5.**
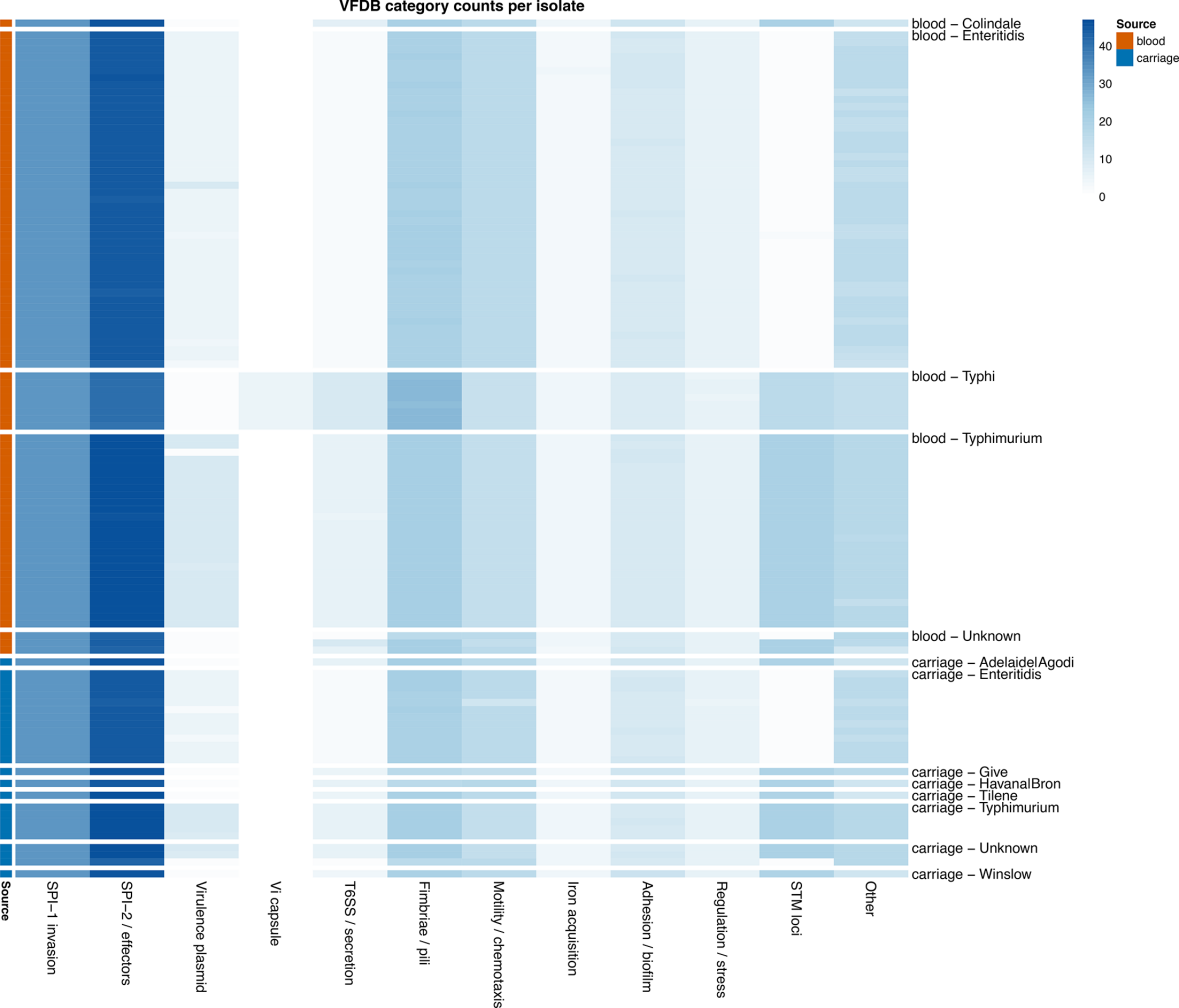
Virulence-associated gene category profiles among bloodstream and carriage-associated *Salmonella* isolates. Heatmap showing counts of virulence-associated genes identified from VFDB annotations, grouped into functional categories: SPI-1 invasion, SPI-2 effectors, virulence plasmid-associated genes, fimbriae/pili, motility/chemotaxis, iron acquisition, adhesion/biofilm, regulation/stress response, Vi capsule, T6SS/secretion and STM loci. Rows represent individual isolates and are grouped by source and serovar, with bloodstream isolates indicated in orange and carriage isolates in blue. Columns represent the total number of genes detected within each virulence category for each isolate. Colour intensity reflects increasing gene counts from low (white) to high (dark blue).

In total, 238 putative virulence factors identified using VFDB were grouped into 12 biological categories. There were no differences observed in the number of virulence-associated genes detected (Fig. 5); however, there were fewer rectal carriage isolates compared to iNTS BSI isolates in the dataset. The main differences observed were associated with serovar rather than source. For example, a larger number of fimbriae-/pili-associated virulence genes were detected in *S*. Typhi compared to other *S. enterica* serovars, whereas the largest number of plasmid-associated virulence genes were detected in *S*. Typhimurium isolates (Fig. 5).

From 26 genomes drafted utilizing long reads, 18 plasmids containing the core *spv* virulence locus were reconstructed. From these, 14 were ~60 kp non-mobilizable IncI plasmids from *S*. Enteritidis genomes (4 rectal carriage, 10 BSI isolates), including one case whereby a genomically similar virulence plasmid was detected from a rectal carriage and BSI *S*. Enteritidis genome (patient ID 20788). All 14 plasmids irrespective of source (BSI vs. rectal carriage) carried *bla*_TEM-1_ ARG in addition to *spvBCD* and *mig-5*, which may provide a competitive advantage. In four *S*. Typhimurium genomes, a ~110 kb *in silico* predicted conjugative *spv*-containing IncF plasmid was detected (Data S3). These plasmids originated from genomes from *Salmonella* isolates recovered from two children (20399 and 20722), where both the BSI isolate and the corresponding rectal carriage isolate contained a genomically similar virulence plasmid. A larger number of virulence-associated and ARG were detected on all the ~110 kb IncF plasmids with *spvBCD* present alongside *pefABCD* genes, often associated with fimbriae and adherence, and *rck* genes associated with complement killing and invasion mechanisms [43]. In addition to multiple virulence-associated genes, eight ARG capable of conferring resistance to multiple antibiotics were detected (*aph3*, *aph6*, *aadA*, *catA*, *dfraA*, *bla*_TEM-1_, *sul1* and *sul2*).

### Concomitant *Salmonella* Carriage and BSI

Rectal swabs collected at admission from 232 children (58 iNTS BSI cases, 174 controls) were screened. *Salmonella* was detected in 44/58 (76%) BSI cases vs. 7/174 (4%) controls (Table 2), corresponding to a relative risk of 18.86 (CI 9.00–39.53, *χ*²=126.7, *P*<0.0001), indicating that children with a confirmed *Salmonella* BSI were >18 times more likely to be colonized with *Salmonella* compared to those without a confirmed BSI (Table 2, Fig. S3).

**Table 2.** Relative risk (risk ratio) and chi-squared to examine the relationship between a *Salmonella* BSI and *Salmonella* concomitant intestinal carriage

Group	Values	Risk or RR	*P*-value
Exposed: *Salmonella* BSI	44/58	0.76	–
Unexposed: no BSI	7/174	0.04	–
Risk ratio	–	18.86 (CI 9.00–39.53)	
Chi-squared	–	X² = 126.7	<0.0001

Among the seven colonized controls (children without a BSI), WGS was possible for four isolates, which represented distinct serovars Winslow, Adelaide/Agodi, Give and Havana/Bron (Data S3, where serovar identification was inconclusive; both possibilities were reported). Only the Havana/Bron genome carried ARG (with *dfraA14*, *qnrB1*, *aac(6′)-Ib-cr5*, *bla_OXA_*_-1_, *catB3*, *tet*(A), *aac(3)-IIe*, *aadA1* and *catA1*, all on a 283 kb IncHI2 plasmid). No differences in ARG carriage were observed by time of collection (admission, hospitalization or discharge).

### Genomic relatedness between intestinal and BSI *Salmonella enterica*

Between 2018–2020 and 2024–2025, REP-PCR was used to screen up to five colonies per rectal swab. Of 44 paired cases, 20/40 (50%) showed a matching REP profile (Fig. S4), from 19 children (1 with 2 BSI isolates). Four of these were HAI; the rest were CAI. Isolates with a matching REP-PCR profile between intestinal carriage and BSI were subject to WGS, subject to viability upon re-culture. WGS was available for *S. enterica* isolates from 13 patients, of whom 11 had the same species/serovar/ST in blood and rectal isolates. Of the 11 children with matching *S. enterica* STs recovered from both blood and rectal swabs, 3 had an *S*. Typhimurium HAI, whereas 8 children with a CAI had *S*. Enteritidis in both their blood cultures and rectal microbiota.

**Fig. 6.**
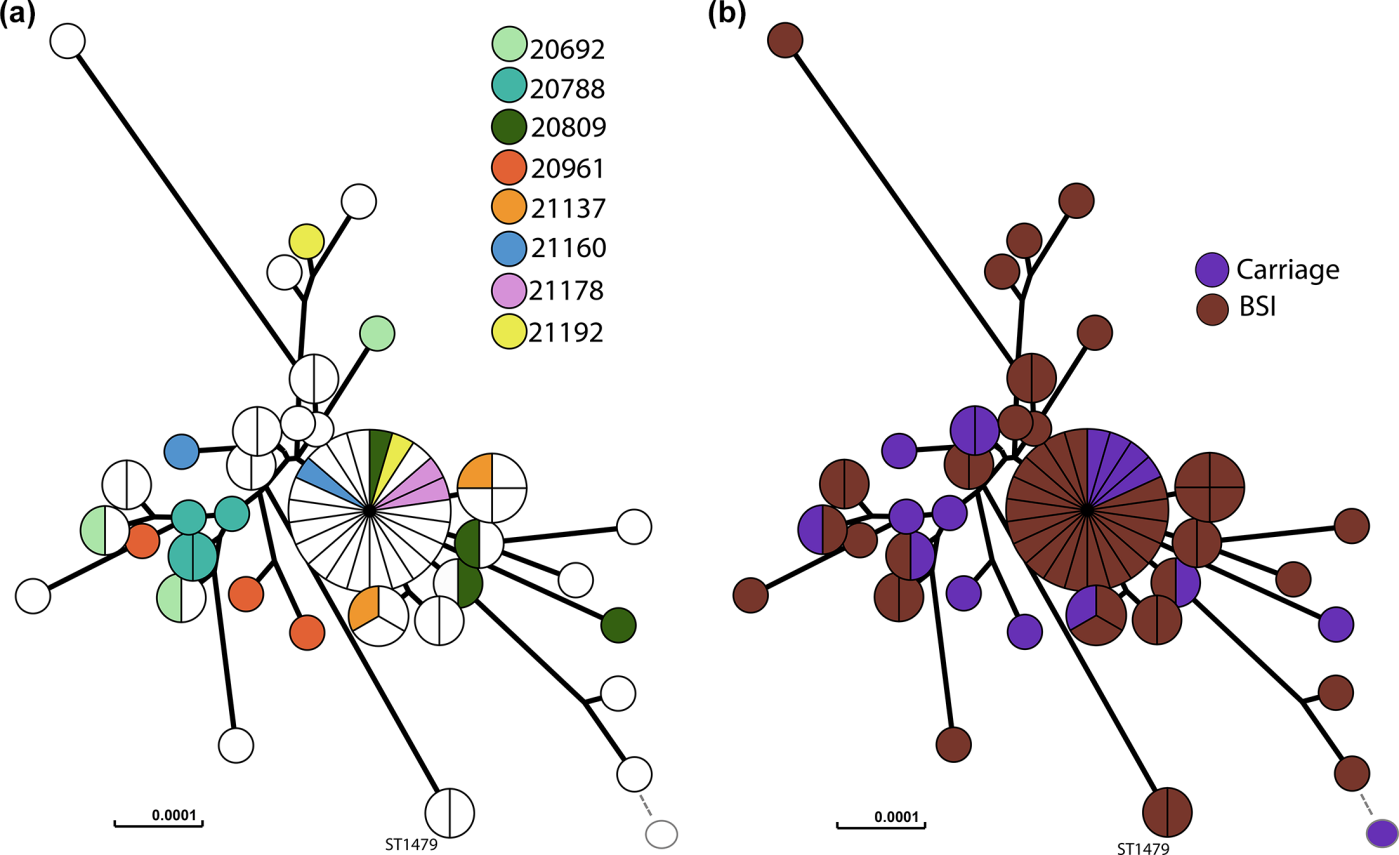
Genomic clustering analysis of *S*. Enteritidis ST11 genomes. (a) Nodes representing individual genomes were coloured according to patient ID for those patients with corresponding ST match isolates cultured from rectal swabs (carriage) and blood cultures (BSI), (b) nodes representing individual genomes were coloured according to the source of sample: carriage or BSI.

Genomic diversity within an ST was observed, with isolates of both ST11 and a single-allele-difference ST7945 forming clusters (Figs 6 and S5). The two ST1479 isolates clustered separately, and the longest distant branch was observed for an ST11 *S*. Enteritidis assembled from long reads only and may include erroneous sequences distorting the clustering (Figs 6 and S5, Data S4). Short-read SNP analysis of ST11 *S*. Enteritidis revealed that genomically similar strains (0–3 SNPs) were detected between the rectal carriage and BSI isolates for four patients (20692, 20788, 21137 and 21160). Additionally, for the cluster of ST7945, all pairs from three patients were within three SNPs, with a maximum SNP distance of 1 for each combination of rectal carriage – BSI *Salmonella* genome within the same child, suggesting the presence of the same (Data S4).

**Fig. 7.**
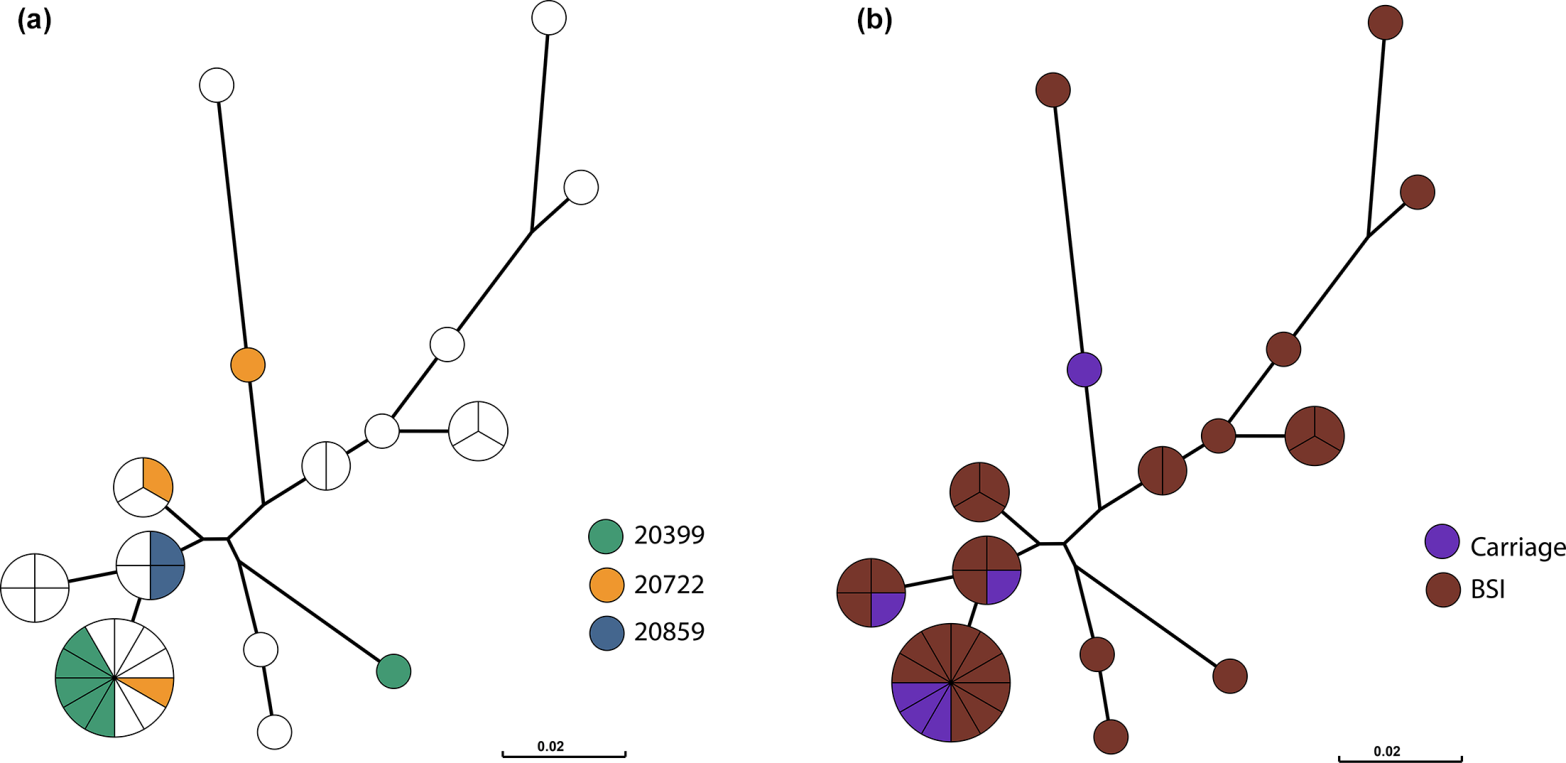
Genomic clustering analysis of *S*. Typhimurium ST313 genomes. (a) Nodes representing individual genomes were coloured according to patient ID for those patients with corresponding ST match isolates cultured from rectal swabs (carriage) and blood cultures (BSI), (b) nodes representing individual genomes were coloured according to the source of sample: carriage or BSI.

*S*. Typhimurium clustering revealed diversity within ST313, with three sub-groups containing isolates from BSI and carriage (Figs 7 and S5). Although clustering of BSI and carriage isolates to the same sub-group occurred for patients 20859 and 20399 (Figs 7 and S5, Data S4), genomes from other children with a BSI clustered together. This was mirrored in complementary SNP analysis, which also suggests the same strain was identified from BSI and rectal carriage samples for patients 20859 and 20399 (all within two SNPs, Fig S5, Data S4). Further, a rectal carriage isolate from patient 20722 was identical (0 SNPs) to four BSI and rectal carriage isolates from patient 20399 (Fig. S5, Data S4). Clustering both within and across patients suggests circulation of a limited number of endemic clones, making it difficult to disentangle transmission dynamics or to distinguish carriage from infection sources.

## Discussion

In this cohort of children with complicated SAM, most *Salmonella* BSIs were CA, consistent with existing evidence that *Salmonella* is a leading cause of BSI in African children [20,21, 58,60]. Similar findings have been described in Niger, where Médecins Sans Frontières has previously reported *Salmonella* BSI in SAM patients [29,61]. Risk factors for BSI include SAM itself, HIV, malaria and poor WASH conditions [28,62,64].

Nutrition shapes the gut microbiota, and SAM may increase the abundance of *Enterobacterales* [65,67]. In immunocompromised hosts, intestinal carriage can serve as a reservoir for invasive infection [68,70]. Our findings show *Salmonella* carriage to be strongly associated with concurrent BSI, with genomically similar strains identified in blood and rectal isolates from 11 of 13 children (where WGS data are available). However, clustering and SNP analyses both indicate that there are likely multiple circulating lineages in the community, making it difficult to infer direct progression from carriage to infection in individual cases. In contrast, *Salmonella* recovered from controls without BSI were diverse and distinct from invasive strains. Similar work by Gu *et al.* [71] in critically ill patients demonstrated that BSI strains can sometimes be traced to the gut, though often non-identical [71], indicating that they were able to track the BSI strain in the gut for a proportion of patients only.

The global burden of AMR is substantial, with an estimated 4.71 million deaths associated with bacterial AMR in 2021 [72]. Modelling projections suggest that up to 8.22 million deaths could be associated with AMR globally by 2050 [72]. Malnourished children are particularly vulnerable to poor outcomes from AMR infections [65], and *Salmonella* is listed as a critical priority pathogen by the WHO [73]. Historically, co-trimoxazole, ampicillin and chloramphenicol were first-line treatments for enteric and iNTS infections in sub-Saharan Africa [66]. Rising resistance has driven the use of cephalosporins and fluoroquinolones, but this has accelerated the carriage and spread of MGEs and extended-spectrum *β*-lactamase [66]. In Niger, where access to broad spectrum or second-/third-line antibiotics with activity against MDR organisms is limited, effective treatment is increasingly constrained. In our cohort, most children received ceftriaxone or penicillin and gentamicin, as reported previously [29].

ST313 *S*. Typhimurium, which is widespread across sub-Saharan Africa and frequently MDR [74], accounted for one-third of *Salmonella* BSI in our study. Isolates were mostly resistant to penicillins, likely due to the presence of *bla*_TEM-1_, often found co-localized on plasmids harbouring multiple virulence genes. ST313 has been consistently linked to invasive disease [74,77] with evidence of persistent intestinal carriage after BSI and shared strains within households [76]. Our study also identified ST11 and ST7945 *S*. Enteritidis (the latter being a local single-locus variant), highlighting the dominance of a few lineages across iNTS cases. Although the dataset is limited, ST7945 *S*. Enteritidis was most apparent in dry seasons, re-emerging after the 2017 rainy season, which may indicate different transmission routes and/or enhanced survival mechanisms in dry environmental conditions. *S*. Typhi BSI (all CA) were also observed, with genomic diversity across clades 2.2, 2.3.1 and 3.1.1, consistent with isolates from Nigeria [78]. Available data suggest long-term regional circulation of diverse *Salmonella* clones. Mbuyi-Kalonji *et al*. found an NTS carriage rate of 4.4% (*n*=98/2234) in the Democratic Republic of Congo and found genetically similar isolates to BSI in members of the same community [79], further evidencing that *Salmonella* is endemic within West Africa.

There were limitations in our study. Up to five putative *Salmonella* colonies were selected from rectal samples for differentiation to compare to corresponding BSI *Salmonella*; however, this approach was not performed locally in Niger. It was not feasible to perform culture and molecular typing on multiple colonies from *Salmonella* isolated from blood cultures. No pre-enrolment samples were collected, limiting insights into carriage dynamics before hospitalization. The lack of post-discharge follow-up prevented assessment of persistence or shedding following discharge from inpatient care. It was not possible to determine the total number of children presenting with suspected malaria or febrile illnesses during this study. Laboratory analyses were retrospective and spanned two periods, though positive controls confirmed reproducibility of *Salmonella* recovery across both time periods. Freezer archives did not contain 100% of rectal microbiota, and 58 admission samples from children who had an iNTS BSI were available from a total of *n*=67. Finally, as an observational study, causal pathways between carriage and BSI cannot be firmly established. Future clinical studies are needed to specifically evaluate the effectiveness of carriage and BSI risk to determine predictive value to make the risk–prediction pathway clearer.

Additionally, future work should investigate the microbiome before and during malnutrition to better understand dysbiosis and pathogen overgrowth. Longitudinal studies combining stool and blood genomics in community settings will be essential to clarify whether carriage predicts BSI risk. Our data suggest that faecal carriage occurs alongside invasive *Salmonella* in many children with complicated SAM, but it remains unclear whether carriage precedes infection, results from haematogenous spread or reflects simultaneous acquisition [80,84]. There have been reports indicating an association between a *Salmonella* infection and concurrent colonization, which is higher in children with BSI compared to healthy children~1–2.5% [81].

In conclusion, most BSIs in this cohort of children with complicated SAM were caused by *Salmonella*, predominantly ST313 *S*. Typhimurium and ST11/ST7945 *S*. Enteritidis. Many cases were accompanied by faecal carriage of related strains, suggesting endogenous infection risk. In resource-limited settings, where WASH deficits drive transmission and access to diagnostics is constrained, affordable gut or rectal screening point-of-care tests paired with context-specific clinical management plans could help identify children at highest risk of BSI. Such approaches could be developed to reduce the burden of invasive *Salmonella* in vulnerable populations.

## Supplementary material

10.1099/mgen.0.001788 Supplementary Material 1.

10.1099/mgen.0.001788 Supplementary Material 2.

## List of abbreviations

AMR: antimicrobial resistance
ARG: antibiotic resistance gene
BSI: bloodstream infection
CA: community-acquired
CAI: community-acquired infection
HA: healthcare-acquired
HAI: healthcare-associated infection
iNTS: invasive non-typhoid *Salmonella*
MDR: multidrug-resistant
MIC: minimum inhibitory concentration
MLST: multilocus sequence type
NTS: non-typhi *Salmonella*
REP-PCR: repetitive element sequence-based PCR
SAM: severe acute malnutrition
ST: sequence type
UKHSA: UK Health Security Agency
WASH: water, sanitation and hygiene
WGS: whole-genome sequencing
WHO: World Health Organization
XLD: xylose lysine deoxycholate
