# Supplementary material for: Salmonella bloodstream infection and concurrent intestinal colonization in children with severe acute malnutrition in Niger
Source: Microb Genom. 2026 Jul 20;12(7):001788. doi: 10.1099/mgen.0.001788 (PMC13384236; doi:10.1099/mgen.0.001788)
Supplement: Supplementary Material 1. [file mgen-12-01788-s001.pdf]

Figures S1 and S2 summarise the Poppunk model fitting analysis performed using the standard PopPUNK workflow. Alternative clustering approaches were compared to identify the most appropriate partitioning of within-lineage and between-lineage relationships. The BGMM and HDBSCAN-based refinement approaches were evaluated, and model performance was assessed using fit statistics, network structure, and cluster stability metrics. The final model was selected based on optimal separation of within-strain and between-strain distances. Core distance distributions were inspected to ensure clear discrimination between closely related and divergent isolates.

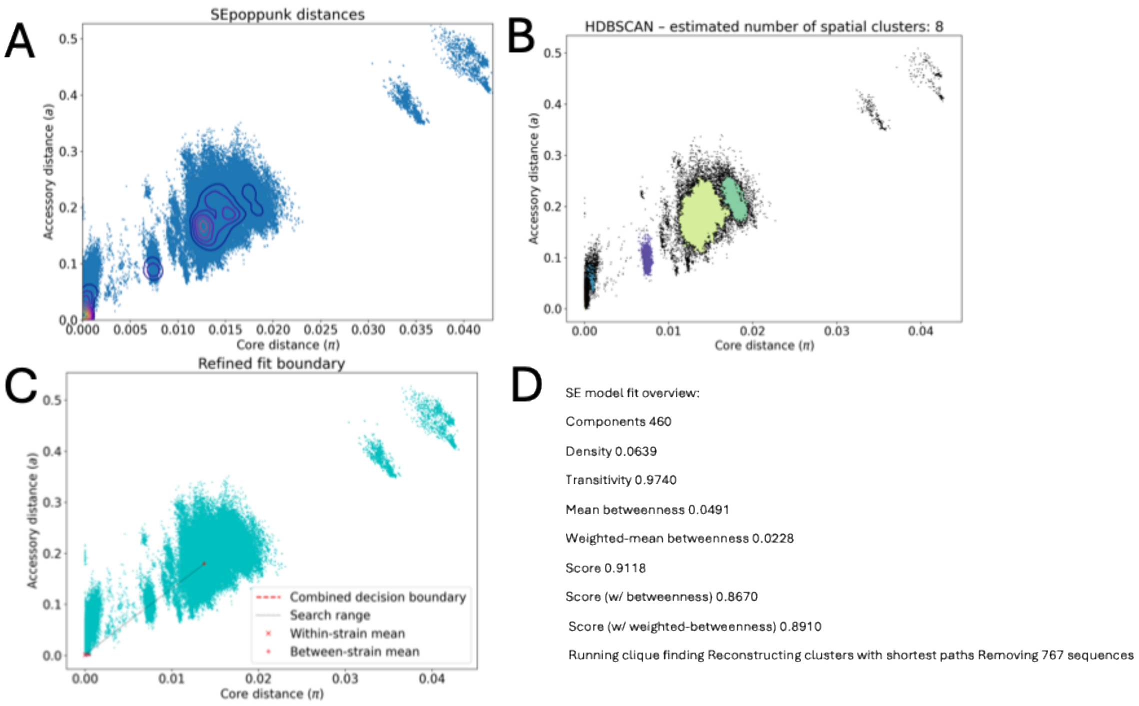

S1 Fig – *Salmonella enterica* serovar Enteritidis poppunk model development: A - S. Enteritidis poppunk database distance distribution, B – HDBSCAN model fit, C – model refinement, D, model fit overview metrics. The core distance π, is the genetic distance based on shared core genome content. The Accessory distance a is the distance based on accessory genome content (i.e., presence/absence of accessory genes). The pairwise comparison between genomes is shown and coloured in B according to assignment cluster. Black dots represent genomes unassigned.

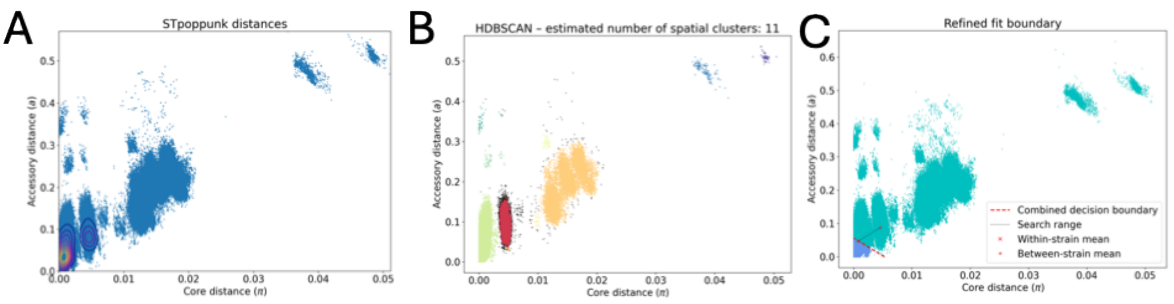

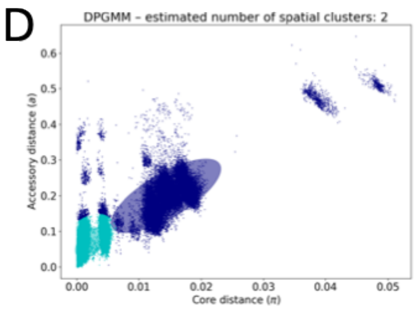

E

	HDBSCAN	BGMM
Components	114	68
Density	0.3492	0.5175
Transitivity	0.8575	0.9869
Mean betweenness	0.1321	0.0372
Weighted-mean betweenness	0.027	0.0074
Score	0.5581	4762
Score (w/betweenness)	0.4844	0.4585
Score (w/ weighted-betweenness)	0.543	4727
Removing sequences	2154	345

S2 Fig – *Salmonella enterica* serovar Typhimurium poppunk model development: A - S. Typhimurium poppunk database distance distribution, B – HDBSCAN model fit, C – model refinement, D BGMM model fit,E model fit overview metrics. The core distance π, is the genetic distance based on shared core genome content. The Accessory distance a is the distance based on accessory genome content (i.e., presence/absence of accessory genes). The pairwise comparison between genomes is shown and coloured in B according to assignment cluster. Black dots represent genomes unassigned. For *Salmonella enterica* serovar Typhimurium, the HDBSCAN produced a suboptimal model fit and removed a high number of sequences. The data was re-fit to the BGMM model (K=2) for use in the study.

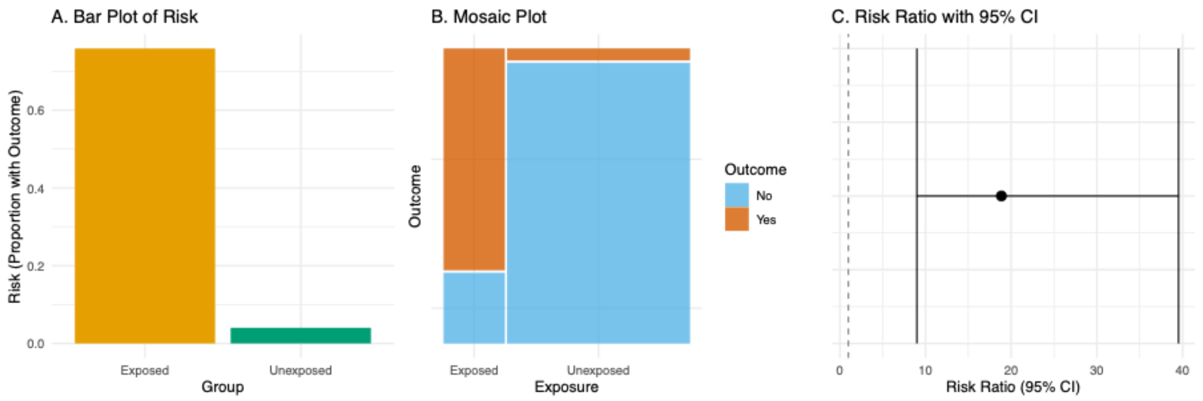

S3 Fig - A) Graphical representation of the statistical analysis determining the relative risk between *Salmonella* carriage and a confirmed BSI. Bar plot displaying the estimated risk ratios across study groups. B) Mosaic plot showing the distribution of observations in the dataset. C) Graphical representation of risk ratios with corresponding 95% confidence intervals.

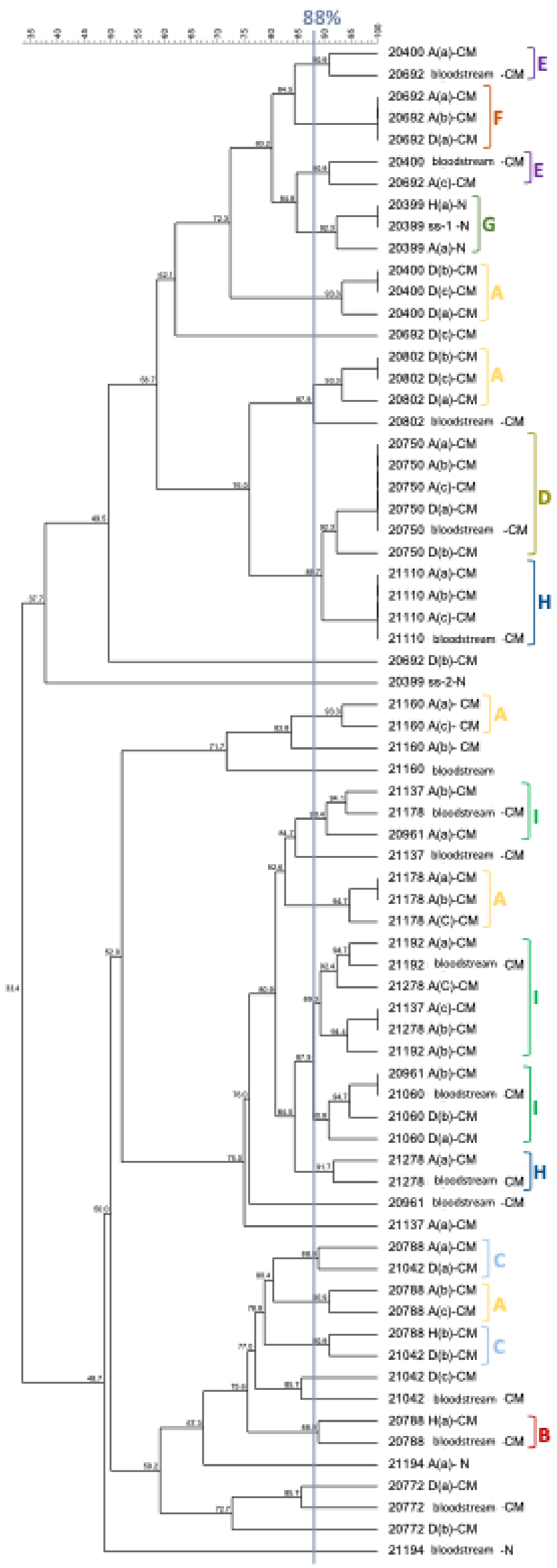

A - Isolates collected from the same rectal swab, similar profile, potential duplication.

B - BSI profile matches to isolates purified from corresponding rectal swab collected during hospital stay.

C – Isolates cultured from different patient's rectal swabs with similar genetic profile.

D – BSI profile matches to isolates purified from corresponding rectal swabs collected at admission and discharge.

E – BSI profile from one patient with similar genetic profile from a different patient's rectal swab isolate collected at admission.

F – Isolates purified from rectal swabs collected at admission and discharge with similar genetic profile.

G – BSI profile matches to isolates purified from corresponding patient rectal swabs collected at admission and patient's deterioration.

H – BSI profile matches to isolates purified from corresponding patient's rectal swab collected upon admission.

I - A patient's BSI matches with isolates from different patient's rectal swabs collected at either admission, deterioration or discharge.

Number of children	Category	Patient IDs
5	A	20400, 20802, 21160,21178, 20788
1	B	20788
2	C	20788, 21042
1	D	20750
2	E	20400, 20692
1	F	20692
1	G	20399
2	H	21110, 21278
5	I	21137, 21178, 20961, 21192, 21060

S4 Fig – REP-PCR dendogram of *Salmonella* colonies isolated from rectal swabs and from blood cultures. Isolates with a match >88% were considered for whole genome sequencing and further analysis.

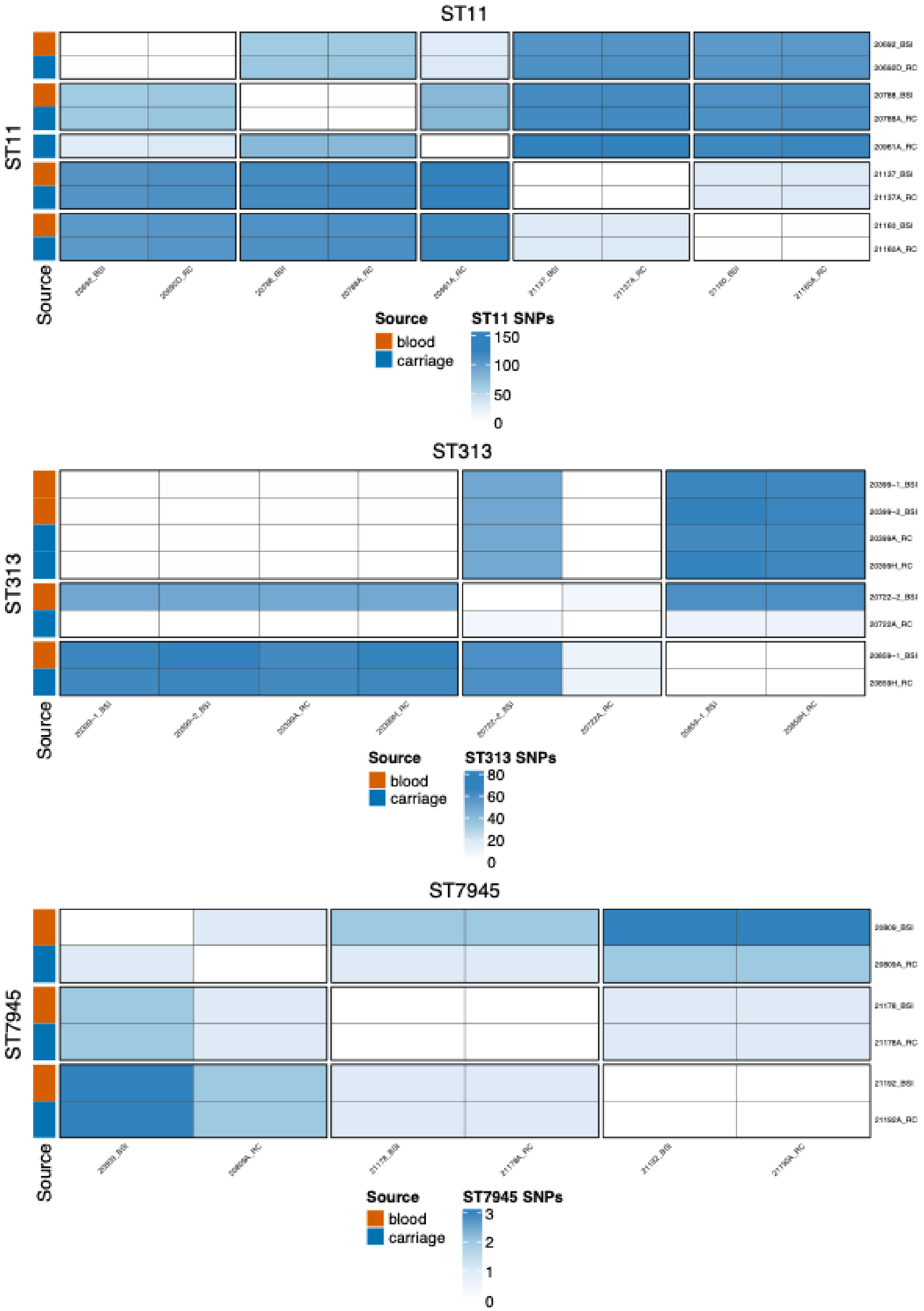

S5 Fig – Pairwise SNP distance heatmaps generated for *Salmonella enterica* serovars Enteritidis (ST313) and Typhimurium ST11, ST7945), derived from snippy core aligments. The source of each isolate (blood = BSI and carriage = rectal carriage) is appended to each heatmap. The intensity of the blue matrix indicates a larger number of pairwise SNPs between the isolates.
